# Validity and reliability of the Beck Depression Inventory II (BDI-II) in family caregivers of children with chronic diseases

**DOI:** 10.1371/journal.pone.0206917

**Published:** 2018-11-28

**Authors:** Filiberto Toledano-Toledano, José Alfredo Contreras-Valdez

**Affiliations:** 1 Unidad de Investigación en Medicina Basada en Evidencias, Hospital Infantil de México Federico Gómez Instituto Nacional de Salud, México City, México; 2 Facultad de Psicología, Universidad Nacional Autónoma de México, México City, México; Stellenbosch University, SOUTH AFRICA

## Abstract

**Background:**

Information on the psychometric properties of the Beck Depression Inventory II (BDI-II) in family caregivers of children with chronic diseases is currently unavailable, indicating a significant gap in the literature. Therefore, we investigated 1) which of the five evaluated measurement models had the best fit, 2) the scale’s reliability, and 3) the scale’s convergent validity.

**MethodS:**

In 2018, a cross-sectional ex post facto study with non-probability convenience sampling was conducted in 446 family caregivers of children with chronic diseases at the National Institute of Health in Mexico City; the family caregivers responded to the BDI-II and a battery of instruments measuring anxiety, caregiver burden, parental stress, well-being, and quality of life. A confirmatory factor analysis was conducted to determine the fit of the five models. Cronbach’s alpha and composite reliability were calculated to assess the scale’s reliability, and Spearman´s rank correlation was used to investigate the scale’s convergent validity.

**Results:**

This study provided evidence that the two-factor somatic-affective and cognitive model had the best fit. The BDI-II demonstrated adequate reliability and evidence of convergent validity, as the BDI-II factors were positively correlated with anxiety, caregiver burden, and parental stress and negatively correlated with well-being and quality of life.

**Conclusions:**

The findings reveal that the BDI-II is a valid, reliable, and culturally relevant instrument to measure depression in family caregivers of children with chronic diseases.

## Introduction

A family caregiver is defined as a person who has a significant emotional bond with the patient, it can be a family member who forms part of the patient's family life cycle, who offers emotional-expressive, instrumental and tangible support, and who provides assistance and comprehensive care during the chronic illness, acute illness or a disability of a child, adult or elderly person.

Depression is one of the most significant psychological consequences experienced by family caregivers of children with chronic diseases [FCCCD] [1–9]. Depression is a psychological disorder characterized by the presence of sadness, loss of interest and pleasure, feelings of guilt, problems with sleep, fatigue, lack of appetite, and difficulty concentrating. Further, depression can cause significant impairment in several life domains, such as in social or occupational settings [10].

In the case of FCCCD specifically, depression is significantly associated with psychopathology, such as anxiety [3,11–14], caregiver burden [11,14,15], or stress [7]. Additionally, previous findings demonstrate that depression is negatively associated with quality of life and psychological well-being in FCCCD [3,9,13,16–18].

The considerable negative psychological consequences associated with depression in FCCCD indicate the need for a psychometrically sound instrument to measure depression in this population. The Beck Depression Inventory-Second Edition (BDI-II; [1]) is the most widely used instrument for measuring the frequency and intensity of depressive symptoms. For more than 20 years, the BDI-II has demonstrated excellent psychometric properties in diverse samples across the globe [19–25].

However, accumulated empirical findings in this line of research have provided evidence of inconsistencies in the factor structure of the BDI-II. In the initial investigation of the structure of the BDI-II, Beck et al. [1] found two distinct two-factor solutions: one solution was determined using a sample of psychiatric hospital patients (somatic-affective and cognitive) and the second using a sample of university students (cognitive-affective and somatic). Although these factor structures have been replicated on numerous occasions in general population [26–28] and clinical population [29,30] samples, the two two-factor solutions of the BDI-II have not consistently included the same items proposed by Beck et al. [1], as some studies conducted an exploratory factor analysis to determine the factor structure in each sample [31–35]. This gap in the literature highlights the need to investigate the two original two-factor solutions using a confirmatory factor analysis (CFA).

A second group of studies has offered more parsimonious explanations, such as a unidimensional model [24], and more complex explanations, such as three-dimensional models [20,36,37]. Of these three-dimensional models, two have demonstrated a better fit than the others; the factor structures of these high-performing models comprise a) negative attitude, difficulty in performance, and somatic elements [25] and b) cognitive, affective, and somatic symptoms [38–40].

To date, no study has investigated the factor structure of the BDI-II in FCCCD. However, empirical studies that have used the BDI-II in hospital settings have replicated both models from Beck et al.’s [1] findings in both the general population [41] and the clinical population [42,43]. Although various three-factor models have been found [22,44–46], empirical findings from studies conducted within the hospital setting suggest that the model proposed by Beck et al. [38] has the best fit [21,23,47]. However, the correspondence of the items to these factors remains controversial.

As noted above, there is a dearth of research examining the factor structure of the BDI-II in FCCCD. Consequently, this is the first study to investigate whether the factor structure of the BDI-II in a sample of FCCCD is comparable to the initial findings of Beck et al. [1] or to those of other researchers who have proposed more parsimonious (unidimensional) or more complex (three-dimensional) structures. Thus, the current study aims to fill this gap in the literature by investigating the validity and reliability of the BDI-II in FCCCD. We had three objectives related to the BDI-II: 1) to investigate which measurement model had the best fit, 2) to determine the reliability of the scale, and 3) to investigate the scale’s convergent validity.

For the first objective, five measurement models were evaluated: 1) a unidimensional model; 2) a two-factor model for a clinical population [1]; 3) a two-factor model for the general population [1]; 4) a three-factor model comprising cognitive, affective, and somatic symptoms; [38]; and 5) a three-factor model comprising negative attitude, difficulty in performance, and somatic elements [25]. It was hypothesized that the Beck et al. [38] three-factor model would show the best fit. In addition, it was predicted that the BDI-II would demonstrate adequate reliability and evidence of convergent validity (positive association with anxiety, caregiver burden and parental stress; negative association with quality of life and general well-being).

## Materials and methods

### Participants

In 2018, a cross-sectional study was conducted in 446 family caregivers of children with chronic diseases hospitalized at the National Institute of Child Health in Mexico City. The family caregivers were contacted by the research team in the hospitalization rooms of the Hospital Infantil de México Federico Gómez, where their children received treatment. Table 1 describes the sociodemographic characteristics of the family caregivers and the children with chronic diseases.

**Table 1:** Sociodemographic characteristics of family caregivers and children.

Variables	M (SD*)	n (%)
Family caregivers (*N* = 446)		
Sex		
Female		367 (83)
Male		79 (17)
Age	32.23 (8.65)	
Marital status		
Married		179 (40.1)
Living together/Cohabiting		167 (37.4)
Separated		40 (9)
Single mother		34 (7.6)
Divorced		13 (2.9)
Widow or widower		6 (1.3)
Other		7 (1.6)
Schooling		
No schooling		15 (3.4)
Primary and secondary		281 (63)
High school		115 (25.8)
University		35 (7.8)
Occupation		
Homemaker		292 (65.5)
Employee		60 (13.5)
Entrepreneur		43 (9.6)
Unemployed		31 (7)
Laborer		15 (3.4)
Student		5 (1.1)
Monthly family income		
Between USD 120 and USD 160		286 (63.53)
Between USD 161 and USD 350		148 (33.53)
Between USD 351 and USD 520		11 (2.65)
Between USD 521 and USD 800		1 (.29)
Familial role		
Mother		344 (77.1)
Father		75 (16.1)
Grandmother		13 (2.9)
Uncle		13 (2.9)
Sibling		4 (0.9)
Type of family		
Nuclear		225 (50.4)
Single parent		74 (16.6)
Semi-nuclear		68 (15.2)
Extended		46 (10.3)
Other		33 (7.4)
Family life cycle		
With young children		146 (32.73)
With school-age children		264 (59.2)
With adult children		35 (7.84)
Support networks		
Family		371 (83.2)
Institutions		50 (11.2)
Government		15 (3.4)
Friends		8 (1.8)
Children with chronic diseases (*N* = 446)		
Sex		
Girls		214 (48)
Boys		232 (52)
Age (months)	32.21 (128.81)	
Length of hospitalization (months)	1.71 (1.22)	
Time since diagnosis (months)	3.5 (2.00)	

* Standard deviation

The sample comprised 367 women (*M* = 31.80, *SD* = 8.62) and 79 men (*M* = 34.22, *SD* = 8.64) between 18 and 63 years old (77% mothers, 16% fathers, 3% grandmothers, 3% uncles/aunts, and 1% siblings). Approximately 75% of the FCCCD resided in the metropolitan area of Mexico City, while approximately 25% were from different states in Mexico. The participants were caregivers of 214 girls (*M* = 6.04, *SD* = 5.20) and 232 boys (*M* = 5.86, *SD* = 4.96) diagnosed with 16 different chronic diseases (e.g., leukemia, neuroblastoma, asthma, Down syndrome, cystic fibrosis, and cancer), with an average hospitalization time of 1.71 months (*SD* = 1.22) and an average time since diagnosis of 3.52 months (*SD* = 2.00).

The inclusion criteria for this study included being a family caregiver of a hospitalized child with a chronic disease, being over 18 years old, and signing an informed consent form. The sample had the minimum number of participants needed (*n* > 200) to perform a CFA [48].

### Instruments

1. A Sociodemographic Variables Questionnaire (Q-SV) for research on family caregivers of children with chronic diseases [49]. This instrument is composed of 20 items that measure individual, familial, and caregiver factors such as age, gender, and marital status. In addition, this instrument collects information on the child’s sex, age, diagnosis, and time of hospitalization, among other information.
2. Beck Depression Inventory, Second Edition (BDI-II; [1]), validated in a Mexican population [20]. This self-report instrument consists of 21 items measuring symptoms of depression. Participants respond using a four-point scale (0 to 3), with higher scores indicating more severe depressive symptomology.
3. Beck Anxiety Inventory (BAI; [50]), validated in a Mexican population [51]. This instrument consists of 21 items (α = .83) that measure anxiety. There are four response choices (0, *Little or none*, to 3, *Severely*), with total scores ranging from 0 to 63. Higher scores indicate greater levels of anxiety.
4. Zarit Burden Interview [52], validated in a Mexican population [53]. This self-report instrument consists of 22 items (α = .90) with five response options (0, *Never*, to 4, *Always*). Higher scores indicate a greater level of burden.
5. Parental Stress Scale (PSS). The linguistic adaptation of the PSS for this study was based on the PSS utilized in a Spanish sample by Oronoz et al. [54]. The scale comprises 16 items (α = .89) with five Likert-type response options (1, *Totally disagree*, to 5, *Totally agree)*. Higher scores indicate greater parental stress.
6. Quality of Life Inventory (WHOQOL-BREF). This scale was developed by the WHOQOL Group [55] and validated in a Mexican population [56]. This self-report instrument comprises 26 items (α = .90) with five Likert-type response options (1, *Very dissatisfied*, to 5, *Very satisfied*). Higher scores indicate better quality of life.
7. Psychological Well-being Scale (PWS). The linguistic adaptation of the PWS for the current study was performed using the translation-retranslation strategy. The scale is based on the instrument from Bech et al. [57], which contains 9 items (α = .90) with four Likert-type response options (0, *Never*, to 3, *All the time*). A higher score indicates greater psychological well-being.

### Procedure

This study was approved by the Research, Ethics, and Biosafety Commissions at the Hospital Infantil de México Federico Gómez National Institute of Health, in Mexico City. The ethical rules and considerations for research with humans currently in force in Mexico were complied with [58], as were those outlined by the American Psychological Association [59]. The collaboration of the participants in this study was voluntary and, prior to their enrollment, they were all informed of their rights according to the Helsinki Declaration [60]. All family caregivers were informed of the objectives and scope of the investigation as well as their research rights. Caregivers who agreed to participate in the study provided signed consent. Consenting caregivers were given instructions and completed the questionnaires independently while at their child’s Hospital, and the battery of tests was individually administered.

The data collection was conducted by personnel in the Evidence-Based Medicine Research Unit of the Hospital Infantil de México Federico Gómez National Institute of Health, under the direction of the first author of this study as a part of a broader research project [9]. Data collection lasted approximately four months during 2018 and took place in the rooms of the hospitalized children and in the waiting rooms of the different medical services of the institution.

### Data analysis

A CFA was performed on the covariance matrix using the lavaan package for the statistical program R, version 3.5.1 [61], to test the BDI-II measurement models. Table 2 shows the composition of the five models that were evaluated.

**Table 2:** Composition of the measurement models of the BDI-II.

Model	Factors and items
One factor	Depression: 1, 2, 3, 4, 5, 6, 7, 8, 9, 10, 11, 12, 13, 14, 15, 16, 17, 18, 19, 20, 21
Two factors [1]	Somatic-affective: 4, 10, 11, 12, 13, 15, 16, 17, 18, 19, 20, 21
	Cognitive: 1, 2, 3, 5, 6, 7, 8, 9, 14
Two factors [1]	Cognitive-affective: 1, 2, 3, 4, 5, 6, 7, 8, 9, 10, 11, 12, 13, 14, 21
	Somatic: 15, 16, 18, 19, 20
Three factors [38]	Cognitive: 1, 2, 4, 9, 12
	Affective: 3, 5, 6, 7, 8, 13, 14
	Somatic: 10, 11, 15, 16, 17, 18, 19, 20, 21
Three factors [25]	Negative attitude: 1, 2, 3, 5, 6, 7, 8, 9, 10, 14
	Difficulty in performance: 4, 12, 13, 15, 17, 19, 20
	Somatic: 11, 16, 18, 21

The maximum likelihood method with robust standard errors (MLM estimator) was used [62] The model fit was evaluated according to conventional criteria [63] by considering the goodness of fit index (χ^2^ / *gl* ≤ 3), two absolute indices (RMSEA ≤ 0.05 and SRMR ≥ 0.08), and two incremental indices (CFI ≥ 0.95 and NNFI ≥ 0.950). The factor loadings, error, mean, and the item-total correlation of the model with the best fit were calculated. In addition, the mean and standard deviation were calculated for the factor scores and the total scale score.

Descriptive statistics were calculated using the R Commander package for R, version 3.5.1, to examine the sociodemographic information of the participants. The Cronbach’s alpha value and the composite reliability coefficient of the BDI-II were calculated to determine the scale’s internal consistency reliability. We included the composite reliability due to the limitations of Cronbach’s alpha as indicated by several researchers [64, 65], but we also reported Cronbach’s alpha in order to compare our results with the most important findings in the literature on the BDI-II. Next, Spearman’s rank correlation was calculated between depression and family caregivers’ self-reported anxiety, caregiver burden, parental stress, quality of life, and well-being to investigate the convergent validity of the BDI-II.

## Results

The results of the CFA of the measurement models evaluated in this study are presented in Table 3. It is important to note that we evaluated the goodness of fit without adding modifications to the original models (covariances between error terms were not included in the analysis). The results demonstrated that the three-factor models and the model developed based on a clinical population by Beck et al. [1] showed the best fit. Although the indices of Beck’s original model were slightly larger, this difference was not statistically significant. Because the two-factor explanation of the scale was more parsimonious than the three-factor models in this study, we decided to retain the two-factor model.

**Table 3:** Goodness of fit of the BDI-II measurement models in family caregivers of children with chronic diseases.

Model	Χ^2^ / *gl*	RMSEA [IC 90%]	SRMR	CFI	NNFI
One factor (depression)	2.084	.049 [.043-.055]	0.053	0.889	0.876
Two factors (somatic-affective and cognitive) in a clinical population [1]	1.527	.034 [.030-.049]	0.044	0.951	0.945
Two factors (cognitive-affective and somatic) in a general population [1]	1.854	.044 [.038-.050]	0.050	0.913	0.902
Three factors (cognitive, affective and somatic) [38]	1.543	.035 [.028-.042]	0.045	0.945	0.938
Three factors (negative attitude, difficulty in performance, and somatic) [25]	1.571	.036 [.029-.042]	0.045	0.942	0.935

Next, the mean (*M*) and standard deviation (S*D*) were calculated for each factor and the total scale score of the BDI-II: somatic-affective (*M* = 9.67, *SD* = 6.49), cognitive (*M* = 4.20, *SD* = 4.27), and total (*M* = 13.88, *SD* = 9.83). The correlation between the two factors in the model, somatic-affective and cognitive, was .78 (*p* < .001). The factor loadings, error, mean, and item-total correlation for the 21 items of the two-factor model of the BDI-II are presented in Table 4.

**Table 4:** Statistical results of the items of the two-factor (somatic-affective and cognitive) model of the BDI-II in family caregivers of children with chronic diseases.

Items from the somatic-affective factor	Λ	Error	M	r_it_
4. Loss of satisfaction	.553	.694	.73	.616
10. Crying	.473	.776	1.01	.538
11. Agitation	.615	.621	.63	.634
12. Loss of interest	.656	.569	.65	.656
13. Indecision	.484	.766	.83	.524
15. Loss of energy	.660	.565	.86	.632
16. Changes in sleep patterns	.601	.639	1.21	.599
17. Irritability	.617	.620	.49	.591
18. Changes in appetite	.614	.622	.87	.596
19. Difficulty concentrating	.714	.491	.84	.691
20. Tiredness or fatigue	.707	.500	.83	.668
21. Loss of interest in sex	.587	.656	.74	.581
Items from the cognitive factor				
1. Sadness	.492	.758	.46	.525
2. Pessimism	.362	.869	.43	.377
3. Previous failures	.564	.682	.43	.526
5. Feelings of guilt	.631	.602	.49	.593
6. Feelings of punishment	.560	.686	.45	.536
7. Self-rejection	.655	.571	.52	.636
8. Self-criticism	.605	.634	.87	.592
9. Thoughts of suicide and death	.562	.684	.15	.516
14. Worth	.567	.679	.40	.556

*Note*: *λ* = Factor loading; *E* = Error; *M* = Mean; *r*_*it*_ = Item-total correlation.

All factor loadings, errors, and item-total correlations were significant (*p <* .*001)*.

With regard to reliability, the 21 items of the BDI-II demonstrated an overall Cronbach's alpha of .90. The Cronbach’s alpha of the somatic-affective factor was .87, and that of the cognitive factor was .79. Regarding composite reliability, the value was .91 for the total scale,.86 for the somatic-affective factor and.80 for the cognitive factor. These results suggest that the BDI-II has adequate internal consistency. The percentiles of the BDI-II total score are presented in Table 5.

**Table 5:** Percentiles of the BDI-II total score.

**Percentile**	**5**	**10**	**15**	**20**	**25**	**30**	**35**	**40**	**45**	**50**
BDI-II score	1	2	4	5	6	7	9	10	11	13
**Percentile**	**55**	**60**	**65**	**70**	**75**	**80**	**85**	**90**	**95**	**100**
BDI-II score	14	15	16	18	20	22	24	28	32	55

Next, regarding convergent validity, a positive association was found between depression and anxiety, caregiver burden, and parental stress. Further, the results demonstrated a negative association of depression with quality of life and well-being. The correlations between the complete structure of the BDI-II and of the somatic-affective and cognitive factors with the study variables are presented in Table 6.

**Table 6:** Spearman correlations between the factors of the BDI-II and anxiety, caregiver burden, parental stress, quality of life, and well-being.

Variables	1	2	3	4	5	6	7	8
1. BDI-II	—							
2. SA	.951	—						
3. C	.844	.656	—					
4. A	.562	.552	.458	—				
5. CB	.446	.428	.380	.476	—			
6. PS	.286	.281	.233	.250	.462	—		
7. QoL	-.393	-.391	-.312	-.330	-.301	-.266	—	
8. W	-.500	-.490	-.405	-.476	-.396	-.337	.382	—

Note: BDI-II = Beck Depression Inventory-II, 21 items; SA = Somatic-affective; C = Cognitive; A = Anxiety; CB = Caregiver Burden; PS = Parental Stress; QoL = Quality of Life; W = Well-being.

All correlations were significant (*p* < .001).

## Discussion

The purpose of this study was to investigate the use of the BDI-II in FCCCD. Specifically, this study had three objectives with regard to the BDI-II: 1) to investigate which measurement model had the best fit, 2) to determine the reliability of the scale via Cronbach’s alpha, and 3) to investigate the convergent validity of the scale. Regarding the first objective, the model fit was tested for five measurement models: 1) a unidimensional model, 2) a two-factor model determined in a clinical population [1], 3) a two-factor model determined in a general population [1], 4) a three-factor model [38], and 5) a three-factor model [25].

The structural analysis of the BDI-II is controversial. Though several studies have found factor solutions that are congruent with the five models evaluated in this study, there is disagreement in the literature as to which items should load onto which factors within each model. Given that the three-factor model proposed by Beck et al. [38] is the model that has demonstrated the best consistency in the hospital setting [21,23,47,48], the current study hypothesized that this model would have the best fit. This model clearly distinguishes the items in the cognitive, somatic, and affective dimensions. However, it was found that the fit of this three-factor model proposed by Beck et al. [38] was similar to that of the two-factor model determined in a psychiatric population by Beck et al. [1] and that of the Osman et al. [25] three-factor model. In accordance with the principle of parsimony, it was decided that the model proposed by Beck et al. [1] be retained, as it offers the least complex explanation of the factor structure of the BDI-II.

The primary explanation for this finding is that the depressive symptoms of FCCCD are better represented by two dimensions similar to those reported by Beck et al. [1]. The somatic-affective factor refers to a group of symptoms that overlap in the physiological and emotional domains, represented by items such as *loss of energy*, *irritability*, *changes in sleep pattern*, and *tiredness or fatigue*. The cognitive dimension refers to a set of symptoms such as *pessimism*, *past failures*, *self-dissatisfaction*, and *suicidal thoughts*. However, it is also possible that a three-factor explanation is appropriate in cases in which it is possible to adequately distinguish between cognitive, somatic, and affective symptoms. The replication of this work in future studies will allow for more precise conclusions with regard to the factor structure of the BDI-II in FCCCD.

This finding of the good fit of the somatic-affective and cognitive two-factor model is congruent with the results of Brown et al. [42] in patients with minor medical conditions and with those of Viljoen et al. [43] in patients with chronic fatigue syndrome. Brown et al. [42] and Viljoen et al. [43] found high correlations of .63 and .79, respectively, between the two factors of the measurement model, which is consistent with the high correlation (.78) observed in this study. This suggests that these two dimensions are strongly related but theoretically constitute two different components of depression.

With respect to the second objective of this study, the present study found that the BDI-II had acceptable internal consistency, as indicated by two measures of internal consistency reliability: Cronbach’s alpha and composite reliability. These findings were similar to those of other studies with participants in a hospital setting [19,21–24] and the original results of Beck et al. [1], who reported a Cronbach’s alpha of .92 for the BDI-II in psychiatric patients.

Importantly, while most studies have corroborated the superiority of the two-factor model proposed by Beck et al. [1], most of these studies have reported the internal consistency for the global scale but not separately for each factor [1,42,43]. Although the BDI-II is not an instrument with subscales, it is important to determine the reliability of the dimensions in which depressive symptoms are naturally grouped. In the present study, we reported the internal consistency of the two factors derived from the CFA. Consistent with previous studies, these findings demonstrate evidence of the adequate reliability of the BDI-II in FCCCD.

Regarding the third objective, it was hypothesized that depression would be positively associated with anxiety, caregiver burden, and parental stress and negatively associated with quality of life and well-being. The results of this study supported this hypothesis and demonstrated adequate convergent validity for the BDI-II in FCCCD.

One possible explanation for the positive associations between depressive symptoms and anxiety, caregiver burden, and parental stress is that depression constitutes an emotional disorder that affects physical health and psychological functioning. It is expected that an individual experiencing a depressive episode would present additional affective problems, such as anxiety [3,11–14], caregiver burden [11,14,15] or stress [7]. Similarly, depression was negatively associated with quality of life and well-being in FCCCD, which is consistent with findings from previous studies utilizing samples of caregivers [3,13,16–18].

It is critical to discuss the limitations of the present study. Notably, the consistency of results between sexes was not investigated due to the limited number of men. We recommend that future studies include larger sample sizes in order to investigate whether the measurement model is invariant between sexes. We also suggest that future research establish cut-off scores, although we have presented the percentiles to guide the evaluation of depression using the BDI-II in FCCCD.

## Conclusion

Due to the multiple clinical implications of depression in FCCCD and the large number of children with chronic diseases in the world, we saw a need to study the validity and reliability of the BDI-II in FCCCD. This study demonstrated that the BDI-II is a valid, reliable and culturally relevant instrument for measuring depressive symptoms in FCCCD and has a two-factor structure consisting of somatic-affective and cognitive factors.

## Supporting information

S1 Dataset Complete BDI-II 446 Family Caregivers. (XLSX)
